# Ethyl 2-(2-oxo-4-phenyl-2,3-dihydro-1*H*-1,5-benzodiazepin-1-yl)acetate

**DOI:** 10.1107/S1600536810028278

**Published:** 2010-07-21

**Authors:** Daouda Ballo, Noureddine Hamou Ahabchane, Hafid Zouihri, El Mokhtar Essassi, Seik Weng Ng

**Affiliations:** a Laboratoire de Chimie Organique Hétérocyclique, Pôle de Compétences Pharmacochimie, Université Mohammed V-Agdal, BP 1014 Avenue Ibn Batout, Rabat, Morocco; b CNRST Division UATRS, Angle Allal Fassi/FAR, BP 8027 Hay Riad, Rabat, Morocco; c Department of Chemistry, University of Malaya, 50603 Kuala Lumpur, Malaysia

## Abstract

The seven-membered ring in the title compound, C_19_H_18_N_2_O_3_, adopts a boat conformation with the two phenyl­ene C atoms representing the stern and the methyl­ene C atom the prow. The dihedral angle between the best plane through the seven-membered ring (r.m.s deviation = 0.343 Å) and the phenyl substituent is 31.9 (1)°. The dihedral angle between this best plane and the best plane through the eth­oxy­carbonyl­methyl substituent (r.m.s. deviation = 0.058 Å) is 72.2 (1)°.

## Related literature

For the background to 2,3-dihydro-1*H*-1,5-benzodiazepin-2-ones, see: Ahabchane *et al.* (1999 ▶). For a related structure, see: Ballo *et al.* (2010 ▶).
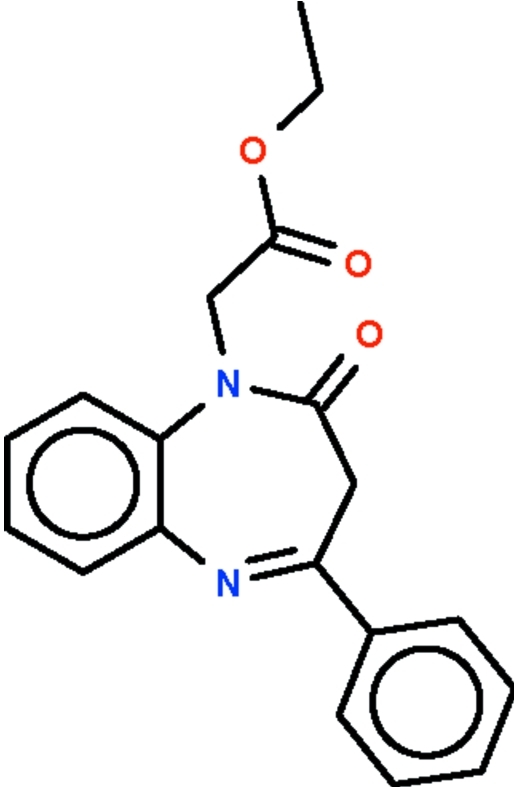


## Experimental

### 

#### Crystal data

C_19_H_18_N_2_O_3_
*M* *_r_* = 322.35
Monoclinic, 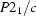

*a* = 12.5198 (4) Å
*b* = 11.7911 (3) Å
*c* = 11.2058 (3) Å
β = 97.843 (2)°
*V* = 1638.75 (8) Å^3^
*Z* = 4
Mo *K*α radiation
μ = 0.09 mm^−1^
*T* = 293 K
0.30 × 0.15 × 0.10 mm

#### Data collection

Bruker X8 APEXII diffractometer
13943 measured reflections
3029 independent reflections
2195 reflections with *I* > 2σ(*I*)
*R*_int_ = 0.033

#### Refinement

*R*[*F* ^2^ > 2σ(*F* ^2^)] = 0.037
*wR*(*F* ^2^) = 0.114
*S* = 1.00
3029 reflections
217 parameters
H-atom parameters constrained
Δρ_max_ = 0.12 e Å^−3^
Δρ_min_ = −0.15 e Å^−3^

### 

Data collection: *APEX2* (Bruker, 2008 ▶); cell refinement: *SAINT* (Bruker, 2008 ▶); data reduction: *SAINT*; program(s) used to solve structure: *SHELXS97* (Sheldrick, 2008 ▶); program(s) used to refine structure: *SHELXL97* (Sheldrick, 2008 ▶); molecular graphics: *X-SEED* (Barbour, 2001 ▶); software used to prepare material for publication: *publCIF* (Westrip, 2010 ▶).

## Supplementary Material

Crystal structure: contains datablocks global, I. DOI: 10.1107/S1600536810028278/zs2051sup1.cif

Structure factors: contains datablocks I. DOI: 10.1107/S1600536810028278/zs2051Isup2.hkl

Additional supplementary materials:  crystallographic information; 3D view; checkCIF report

## Figures and Tables

**Fig. 1.**
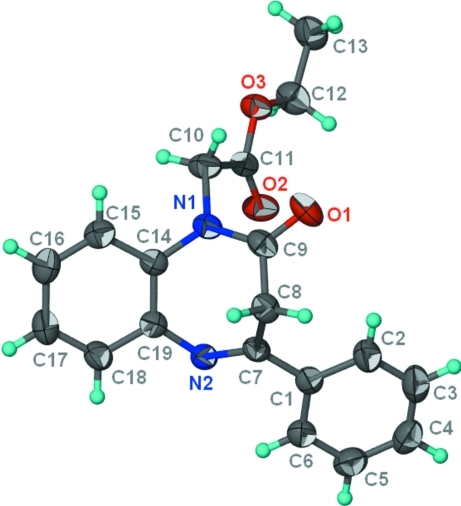
Thermal ellipsoid plot (Barbour, 2001) of the molecule of C19H18N2O3 at the 50% probability level.

## supplementary crystallographic information

### Comment

The background to the class of 2,3-dihydro-1*H*-1,5-benzodiazepin-2-ones is given in an earlier report (Ahabchane *et al.*, 1999). A recent study presents the crystal structure of 1-allyl-4-phenyl-2,3-dihydro-1*H*-1,5-benzodiazepin-2-one (Ballo *et al.*, 2010). The present study has an ethoxycarbonylmethyl group in place of the allyl group (Scheme I, Fig. 1). The principal feature is the seven-membered ring that is fused to a phenylene ring. This ring adopts a boat-shaped conformation, two phenylene carbons representing the stern and the methylene carbon atom the prow [r.m.s deviation 0.343 Å]. The methyl carbon deviates by 0.604 Å from the best plane.

### Experimental

To a solution of 4-phenyl-2,3-dihydro-1*H*-1,5-benzodiazepin-2-one (1 g, 4.2 mmol) in DMF (20 ml) was added ethyl chloroacetate (0.5 g, 4.2 mmol), potassium carbonate (1 g, 7.4 mmol) and a catalytic quantity of tetra-*n-*butylammonium bromide. The mixture was stirred at room temperature for 24 h. The solution was filtered and the solvent removed under reduced pressure. The residue was recrystallized from ethanol to afford the title compound as yellow crystals.

### Refinement

Carbon-bound H-atoms were placed in calculated positions (C–H 0.93–0.97 Å) and were included in the refinement in the riding model approximation, with *U*(H) set to 1.2–1.5*U*_eq_(C).

### Crystal data

**Table:**

C_19_H_18_N_2_O_3_	*F*(000) = 680
*M**_r_* = 322.35	*D*_x_ = 1.307 Mg m^−^^3^
Monoclinic, *P*2_1_/*c*	Mo *K*α radiation, λ = 0.71073 Å
Hall symbol: -P 2ybc	Cell parameters from 3291 reflections
*a* = 12.5198 (4) Å	θ = 2.4–23.1°
*b* = 11.7911 (3) Å	µ = 0.09 mm^−^^1^
*c* = 11.2058 (3) Å	*T* = 293 K
β = 97.843 (2)°	Prism, yellow
*V* = 1638.75 (8) Å^3^	0.30 × 0.15 × 0.10 mm
*Z* = 4	

### Data collection

**Table:**

Bruker X8 APEXII diffractometer	2195 reflections with *I* > 2σ(*I*)
Radiation source: fine-focus sealed tube	*R*_int_ = 0.033
graphite	θ_max_ = 25.5°, θ_min_ = 2.5°
φ and ω scans	*h* = −15→15
13943 measured reflections	*k* = −14→14
3029 independent reflections	*l* = −13→13

### Refinement

**Table:**

Refinement on *F*^2^	Primary atom site location: structure-invariant direct methods
Least-squares matrix: full	Secondary atom site location: difference Fourier map
*R*[*F*^2^ > 2σ(*F*^2^)] = 0.037	Hydrogen site location: inferred from neighbouring sites
*wR*(*F*^2^) = 0.114	H-atom parameters constrained
*S* = 1.00	*w* = 1/[σ^2^(*F*_o_^2^) + (0.0707*P*)^2^] where *P* = (*F*_o_^2^ + 2*F*_c_^2^)/3
3029 reflections	(Δ/σ)_max_ = 0.001
217 parameters	Δρ_max_ = 0.12 e Å^−^^3^
0 restraints	Δρ_min_ = −0.15 e Å^−^^3^

### Fractional atomic coordinates and isotropic or equivalent isotropic displacement parameters (Å^2^)

**Table:**

	*x*	*y*	*z*	*U*_iso_*/*U*_eq_	
O1	0.45383 (9)	0.67927 (10)	0.34251 (11)	0.0662 (4)	
O2	0.43414 (9)	0.64804 (9)	0.06491 (11)	0.0604 (3)	
O3	0.60915 (8)	0.69533 (8)	0.08966 (10)	0.0488 (3)	
N1	0.39095 (10)	0.82128 (10)	0.21765 (12)	0.0460 (3)	
N2	0.16060 (9)	0.76218 (10)	0.14862 (11)	0.0438 (3)	
C1	0.15443 (12)	0.57820 (13)	0.23365 (13)	0.0452 (4)	
C2	0.21662 (14)	0.49224 (14)	0.29222 (16)	0.0599 (5)	
H2	0.2822	0.5097	0.3381	0.072*	
C3	0.18218 (17)	0.38119 (15)	0.28306 (18)	0.0690 (5)	
H3	0.2253	0.3242	0.3214	0.083*	
C4	0.08518 (16)	0.35429 (14)	0.21804 (18)	0.0648 (5)	
H4	0.0618	0.2793	0.2132	0.078*	
C5	0.02215 (14)	0.43823 (15)	0.15974 (18)	0.0631 (5)	
H5	−0.0440	0.4202	0.1156	0.076*	
C6	0.05711 (13)	0.54944 (13)	0.16671 (15)	0.0525 (4)	
H6	0.0147	0.6057	0.1259	0.063*	
C7	0.19264 (11)	0.69709 (12)	0.23782 (13)	0.0423 (4)	
C8	0.27085 (12)	0.73756 (13)	0.34441 (13)	0.0483 (4)	
H8A	0.2497	0.8118	0.3702	0.058*	
H8B	0.2715	0.6853	0.4113	0.058*	
C9	0.38018 (12)	0.74323 (13)	0.30496 (14)	0.0478 (4)	
C10	0.49294 (13)	0.81974 (13)	0.16772 (16)	0.0536 (4)	
H10A	0.5523	0.8288	0.2323	0.064*	
H10B	0.4948	0.8828	0.1124	0.064*	
C11	0.50622 (12)	0.71043 (12)	0.10251 (13)	0.0431 (4)	
C12	0.63354 (14)	0.59312 (14)	0.02606 (16)	0.0592 (5)	
H12A	0.6050	0.5992	−0.0586	0.071*	
H12B	0.6010	0.5277	0.0593	0.071*	
C13	0.75293 (14)	0.58019 (15)	0.04048 (16)	0.0669 (5)	
H13A	0.7712	0.5132	−0.0011	0.100*	
H13B	0.7803	0.5736	0.1245	0.100*	
H13C	0.7843	0.6453	0.0074	0.100*	
C14	0.31063 (11)	0.90246 (12)	0.17520 (12)	0.0414 (4)	
C15	0.34293 (13)	1.01399 (12)	0.15912 (14)	0.0511 (4)	
H15	0.4153	1.0333	0.1776	0.061*	
C16	0.26962 (15)	1.09539 (14)	0.11653 (16)	0.0595 (5)	
H16	0.2924	1.1694	0.1066	0.071*	
C17	0.16234 (15)	1.06815 (14)	0.08833 (15)	0.0596 (5)	
H17	0.1125	1.1238	0.0604	0.072*	
C18	0.12908 (13)	0.95835 (13)	0.10160 (14)	0.0528 (4)	
H18	0.0566	0.9402	0.0810	0.063*	
C19	0.20182 (12)	0.87328 (12)	0.14543 (12)	0.0418 (4)	

### Atomic displacement parameters (Å^2^)

**Table:**

	*U*^11^	*U*^22^	*U*^33^	*U*^12^	*U*^13^	*U*^23^
O1	0.0551 (8)	0.0761 (8)	0.0638 (8)	0.0232 (6)	−0.0050 (6)	0.0014 (6)
O2	0.0439 (7)	0.0632 (7)	0.0729 (8)	−0.0078 (6)	0.0036 (5)	−0.0194 (6)
O3	0.0384 (6)	0.0511 (6)	0.0573 (7)	0.0031 (4)	0.0083 (5)	−0.0115 (5)
N1	0.0373 (7)	0.0501 (7)	0.0512 (8)	0.0040 (5)	0.0081 (6)	−0.0036 (6)
N2	0.0392 (7)	0.0486 (7)	0.0432 (7)	0.0003 (5)	0.0037 (5)	0.0044 (6)
C1	0.0456 (9)	0.0518 (9)	0.0405 (9)	0.0050 (7)	0.0138 (7)	0.0039 (7)
C2	0.0617 (11)	0.0583 (11)	0.0589 (11)	0.0074 (8)	0.0049 (8)	0.0115 (8)
C3	0.0826 (14)	0.0581 (11)	0.0687 (12)	0.0148 (10)	0.0184 (10)	0.0180 (9)
C4	0.0781 (13)	0.0476 (10)	0.0750 (13)	−0.0020 (9)	0.0333 (10)	0.0026 (9)
C5	0.0538 (11)	0.0612 (11)	0.0772 (13)	−0.0070 (8)	0.0189 (9)	−0.0040 (9)
C6	0.0464 (9)	0.0532 (9)	0.0594 (10)	0.0026 (7)	0.0123 (7)	0.0050 (8)
C7	0.0364 (8)	0.0518 (9)	0.0398 (8)	0.0053 (6)	0.0088 (6)	0.0034 (7)
C8	0.0523 (10)	0.0549 (9)	0.0372 (8)	0.0053 (7)	0.0047 (7)	0.0028 (7)
C9	0.0441 (9)	0.0546 (9)	0.0421 (9)	0.0077 (7)	−0.0031 (7)	−0.0086 (7)
C10	0.0390 (9)	0.0538 (9)	0.0696 (11)	−0.0012 (7)	0.0129 (8)	−0.0137 (8)
C11	0.0364 (8)	0.0487 (8)	0.0433 (9)	0.0009 (7)	0.0025 (6)	−0.0019 (7)
C12	0.0610 (11)	0.0597 (10)	0.0570 (11)	0.0105 (8)	0.0082 (8)	−0.0162 (8)
C13	0.0651 (12)	0.0721 (12)	0.0681 (12)	0.0205 (9)	0.0253 (9)	0.0010 (9)
C14	0.0413 (8)	0.0456 (8)	0.0383 (8)	0.0031 (6)	0.0085 (6)	−0.0049 (6)
C15	0.0521 (10)	0.0487 (9)	0.0536 (10)	−0.0049 (7)	0.0108 (7)	−0.0090 (7)
C16	0.0766 (13)	0.0426 (9)	0.0599 (11)	−0.0012 (8)	0.0115 (9)	−0.0004 (8)
C17	0.0711 (12)	0.0516 (10)	0.0552 (10)	0.0119 (9)	0.0053 (9)	0.0088 (8)
C18	0.0480 (9)	0.0592 (10)	0.0498 (10)	0.0056 (7)	0.0019 (7)	0.0078 (8)
C19	0.0430 (8)	0.0473 (8)	0.0349 (8)	0.0009 (7)	0.0050 (6)	−0.0007 (6)

### Geometric parameters (Å, °)

**Table:**

O1—C9	1.2207 (17)	C8—C9	1.496 (2)
O2—C11	1.1955 (17)	C8—H8A	0.9700
O3—C11	1.3281 (17)	C8—H8B	0.9700
O3—C12	1.4537 (17)	C10—C11	1.502 (2)
N1—C9	1.363 (2)	C10—H10A	0.9700
N1—C14	1.4224 (17)	C10—H10B	0.9700
N1—C10	1.4625 (19)	C12—C13	1.489 (2)
N2—C7	1.2805 (18)	C12—H12A	0.9700
N2—C19	1.4102 (18)	C12—H12B	0.9700
C1—C6	1.383 (2)	C13—H13A	0.9600
C1—C2	1.387 (2)	C13—H13B	0.9600
C1—C7	1.480 (2)	C13—H13C	0.9600
C2—C3	1.378 (2)	C14—C15	1.395 (2)
C2—H2	0.9300	C14—C19	1.4005 (19)
C3—C4	1.366 (3)	C15—C16	1.368 (2)
C3—H3	0.9300	C15—H15	0.9300
C4—C5	1.374 (2)	C16—C17	1.375 (2)
C4—H4	0.9300	C16—H16	0.9300
C5—C6	1.381 (2)	C17—C18	1.374 (2)
C5—H5	0.9300	C17—H17	0.9300
C6—H6	0.9300	C18—C19	1.398 (2)
C7—C8	1.515 (2)	C18—H18	0.9300
			
C11—O3—C12	115.86 (12)	C11—C10—H10A	109.5
C9—N1—C14	124.03 (13)	N1—C10—H10B	109.5
C9—N1—C10	116.31 (12)	C11—C10—H10B	109.5
C14—N1—C10	119.65 (12)	H10A—C10—H10B	108.0
C7—N2—C19	119.99 (13)	O2—C11—O3	125.19 (14)
C6—C1—C2	118.26 (15)	O2—C11—C10	124.83 (14)
C6—C1—C7	120.43 (14)	O3—C11—C10	109.95 (12)
C2—C1—C7	121.26 (14)	O3—C12—C13	107.85 (13)
C3—C2—C1	120.61 (17)	O3—C12—H12A	110.1
C3—C2—H2	119.7	C13—C12—H12A	110.1
C1—C2—H2	119.7	O3—C12—H12B	110.1
C4—C3—C2	120.46 (17)	C13—C12—H12B	110.1
C4—C3—H3	119.8	H12A—C12—H12B	108.4
C2—C3—H3	119.8	C12—C13—H13A	109.5
C3—C4—C5	119.83 (17)	C12—C13—H13B	109.5
C3—C4—H4	120.1	H13A—C13—H13B	109.5
C5—C4—H4	120.1	C12—C13—H13C	109.5
C4—C5—C6	119.99 (17)	H13A—C13—H13C	109.5
C4—C5—H5	120.0	H13B—C13—H13C	109.5
C6—C5—H5	120.0	C15—C14—C19	119.38 (13)
C5—C6—C1	120.83 (16)	C15—C14—N1	118.26 (13)
C5—C6—H6	119.6	C19—C14—N1	122.32 (13)
C1—C6—H6	119.6	C16—C15—C14	120.93 (16)
N2—C7—C1	118.53 (13)	C16—C15—H15	119.5
N2—C7—C8	121.78 (13)	C14—C15—H15	119.5
C1—C7—C8	119.65 (13)	C15—C16—C17	120.26 (15)
C9—C8—C7	107.47 (12)	C15—C16—H16	119.9
C9—C8—H8A	110.2	C17—C16—H16	119.9
C7—C8—H8A	110.2	C18—C17—C16	119.74 (16)
C9—C8—H8B	110.2	C18—C17—H17	120.1
C7—C8—H8B	110.2	C16—C17—H17	120.1
H8A—C8—H8B	108.5	C17—C18—C19	121.43 (16)
O1—C9—N1	121.36 (15)	C17—C18—H18	119.3
O1—C9—C8	123.30 (15)	C19—C18—H18	119.3
N1—C9—C8	115.23 (13)	C18—C19—C14	118.24 (13)
N1—C10—C11	110.92 (12)	C18—C19—N2	116.89 (13)
N1—C10—H10A	109.5	C14—C19—N2	124.72 (13)
			
C6—C1—C2—C3	0.4 (2)	C14—N1—C10—C11	−116.19 (14)
C7—C1—C2—C3	−177.05 (15)	C12—O3—C11—O2	−1.0 (2)
C1—C2—C3—C4	−1.3 (3)	C12—O3—C11—C10	−179.08 (14)
C2—C3—C4—C5	1.0 (3)	N1—C10—C11—O2	20.5 (2)
C3—C4—C5—C6	0.2 (3)	N1—C10—C11—O3	−161.39 (13)
C4—C5—C6—C1	−1.1 (3)	C11—O3—C12—C13	−169.68 (13)
C2—C1—C6—C5	0.9 (2)	C9—N1—C14—C15	135.77 (15)
C7—C1—C6—C5	178.28 (15)	C10—N1—C14—C15	−43.45 (18)
C19—N2—C7—C1	−175.16 (12)	C9—N1—C14—C19	−46.5 (2)
C19—N2—C7—C8	2.8 (2)	C10—N1—C14—C19	134.31 (15)
C6—C1—C7—N2	−26.6 (2)	C19—C14—C15—C16	1.0 (2)
C2—C1—C7—N2	150.72 (15)	N1—C14—C15—C16	178.85 (13)
C6—C1—C7—C8	155.40 (14)	C14—C15—C16—C17	−0.2 (2)
C2—C1—C7—C8	−27.3 (2)	C15—C16—C17—C18	−0.8 (3)
N2—C7—C8—C9	−74.71 (17)	C16—C17—C18—C19	1.2 (3)
C1—C7—C8—C9	103.20 (15)	C17—C18—C19—C14	−0.4 (2)
C14—N1—C9—O1	−176.16 (13)	C17—C18—C19—N2	−176.23 (14)
C10—N1—C9—O1	3.1 (2)	C15—C14—C19—C18	−0.7 (2)
C14—N1—C9—C8	7.5 (2)	N1—C14—C19—C18	−178.44 (13)
C10—N1—C9—C8	−173.24 (12)	C15—C14—C19—N2	174.79 (14)
C7—C8—C9—O1	−111.25 (16)	N1—C14—C19—N2	−2.9 (2)
C7—C8—C9—N1	65.00 (16)	C7—N2—C19—C18	−140.71 (15)
C9—N1—C10—C11	64.53 (18)	C7—N2—C19—C14	43.7 (2)
